# Queda da Pressão Arterial Diastólica Durante o Sono tem Maior Poder Preditivo Não Ajustado em Comparação à Queda Sistólica: Um Estudo de Coorte Retrospectivo

**DOI:** 10.36660/abc.20250305

**Published:** 2025-11-18

**Authors:** Mateus de Carvalho Gonçalves, Daniely Santos da Silva, Vitória Nogueira Ribeiro, Vanessa Burgugi Banin, Vanessa dos Santos Silva, Rodrigo Bazan, Silméia Garcia Zanati Bazan, Pasqual Barretti, Luis Cuadrado Martin

**Affiliations:** 1 Universidade Estadual Paulista Júlio Mesquita Filho Faculdade de Medicina Botucatu SP Brasil Universidade Estadual Paulista Júlio Mesquita Filho - Faculdade de Medicina, Botucatu, SP – Brasil

**Keywords:** Hipertensão, Monitorização Ambulatorial da Pressão Arterial, Mortalidade

## Abstract

**Fundamento:**

A queda da pressão arterial (PA) durante o sono é reconhecida como um marcador prognóstico. Poucos estudos avaliaram se a queda da PA sistólica (PAS) ou da PA diastólica (PAD) prediz com maior precisão os desfechos clínicos.

**Objetivo:**

Determinar qual tipo de queda da PA possui maior valor preditivo para desfechos clínicos.

**Métodos:**

Esta coorte retrospectiva avaliou pacientes submetidos à monitorização ambulatorial da PA entre 27 de janeiro de 2004 e 16 de fevereiro de 2012. Os pacientes foram acompanhados até a ocorrência do desfecho primário (óbito por qualquer causa) ou até o fim do período de seguimento (1º de fevereiro de 2022). Curvas de sobrevivência de Cox foram construídas para avaliar qual classificação de queda – PAS ou PAD – melhor distinguiu a ocorrência dos desfechos. A queda foi definida como uma redução noturna da PA entre 10% e 20%. A ausência e a atenuação da queda foram definidas como reduções de ≤ 0% e entre 0% e 10%, respectivamente. A significância estatística foi estabelecida em p < 0,05.

**Resultados:**

Um total de 756 pacientes foi incluído, com idade média de 54 ± 16,4 anos; 42% eram do sexo masculino. Na predição do desfecho primário (mortalidade por todas as causas), a ausência de queda da PAD, ajustada para a PAS média de 24 horas, esteve associada a um *hazard ratio* (HR) de 2,051 [Intervalo de Confiança de 95% (IC95%): 1,147–3,670; p = 0,015). Para o desfecho secundário (mortalidade cardiovascular), a ausência de queda da PAD, também ajustada para a PAS de 24 horas, apresentou um HR de 3,329 (IC95%: 1,317–8,412; p = 0,011). Por outro lado, a ausência de queda da PAS, quando ajustada para a PAS de 24 horas, não apresentou associação estatisticamente significativa com nenhum dos desfechos. No modelo totalmente ajustado – que incluiu idade, diabetes, tabagismo, doença vascular aterosclerótica e doença renal crônica – tanto a queda da PAS quanto da PAD perderam a significância estatística.

**Conclusão:**

A queda da PAD demonstra maior poder preditivo para desfechos clínicos em comparação à queda da PAS, além de contribuir para o valor prognóstico da PAS média de 24 horas (Figura Central).

**Figure:**
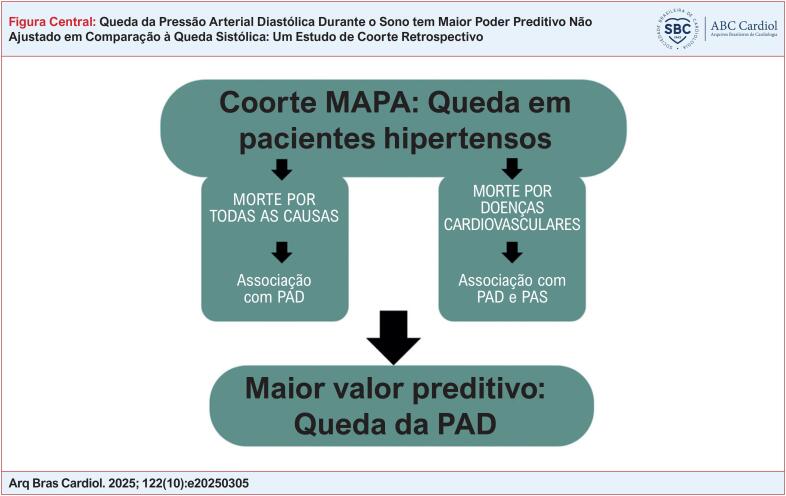


## Introdução

Estima-se que mais de um bilhão de pessoas no mundo tenham hipertensão (1,39 bilhão em 2010).^1^ Entre esses indivíduos, uma grande proporção desconhece o diagnóstico e não toma medidas para controlar essa doença crônica. Isso aumenta as chances de desenvolver doenças cardiovasculares,^2^ como infarto do miocárdio, acidente vascular cerebral e doença renal crônica, que juntas causam cerca de 17,9 milhões de mortes^3^ no mundo a cada ano.

Esses dados ressaltam a relevância da hipertensão para a saúde pública e a necessidade de métodos confiáveis para identificar essa condição. Como a pressão arterial (PA) varia a cada batimento cardíaco,^4^ decisões diagnósticas e terapêuticas não devem ser baseadas em uma única medição. O acesso à média diária da PA é essencial, pois alguns indivíduos podem apresentar níveis hipertensivos durante consultas médicas, mas manter níveis normais em sua rotina diária (hipertensão do avental branco). Ainda, outros podem apresentar níveis normais durante as consultas, mas níveis elevados no dia a dia, o que é chamado de hipertensão mascarada. Essas discrepâncias só podem ser detectadas com precisão fora do ambiente clínico.

Os métodos de aferição da PA fora do ambiente clínico incluem a monitorização ambulatorial da PA (MAPA),^4^ que é capaz de medir a PA tanto durante a vigília quanto durante o sono. Esse exame também indica se há ou não uma redução fisiológica significativa (chamada de "*dipping*") na PA durante o sono. A presença do *dipping* noturno é definida como uma redução da PA entre 10% e 20% em relação aos níveis diurnos. Essa queda está associada a melhores desfechos a longo prazo, independentemente da média de PA nas 24 horas.

Ao analisar os resultados da MAPA, é necessário considerar se o padrão de queda ("*dipping*") está ausente, atenuado, presente ou acentuado (definido como menos de 0%, menos de 10%, entre 10% e 20%, e superior a 20%, respectivamente). Essa classificação pode diferir entre a pressão arterial sistólica (PAS) e a pressão arterial diastólica (PAD), podendo indicar uma redução fisiológica em ambas, apenas na PAD, apenas na PAS ou ausência de redução em ambas. Assim, a diretriz brasileira de MAPA^4^ recomenda que as reduções na PAS e na PAD sejam descritas separadamente no laudo do exame.

No entanto, poucos estudos avaliaram qual tipo de queda – PAS ou PAD – prediz com maior precisão a ocorrência futura de desfechos clínicos. As diretrizes brasileiras^4^ inclusive classificam o nível de evidência dessa recomendação como "D", indicando que se baseia em opinião de especialistas, sem evidências conclusivas.

Assim, o presente estudo teve como objetivo determinar se a queda da PAS, da PAD ou de ambas apresenta maior poder preditivo para a ocorrência de eventos fatais.

## Métodos

Um estudo de coorte retrospectivo foi conduzido para determinar se o poder discriminatório na predição da mortalidade por hipertensão arterial difere entre a queda da PAS e a queda da PAD durante o sono. Foram incluídos pacientes hipertensos em acompanhamento clínico de rotina, com idade acima de 18 anos, que realizaram apenas uma MAPA de 24 horas entre 27 de janeiro de 2004 e 16 de fevereiro de 2012.

A MAPA foi realizada utilizando braçadeiras de tamanho apropriado para cada paciente e seguiu critérios de validação: pelo menos 16 medidas durante o dia e 8 medidas durante o período noturno, realizadas em intervalos de 15 minutos durante a vigília e de 30 minutos durante o sono. Os períodos de sono e vigília foram definidos pelos próprios pacientes, e a monitorização foi realizada em um dia típico.

O acompanhamento teve início a partir da primeira MAPA de 24 horas registrada nos prontuários médicos. Os pacientes foram acompanhados até a ocorrência do desfecho primário (óbito por qualquer causa, avaliado por meio de prontuários médicos ou certidão de óbito) ou até o final do período de acompanhamento (01/02/2022). O desfecho secundário foi a mortalidade cardiovascular (definida como óbito por parada cardíaca súbita, doença isquêmica do coração, insuficiência cardíaca, doença cerebrovascular ou aneurisma de aorta). Foram excluídos pacientes com doença de Alzheimer ou Parkinson, em hemodiálise, receptores de transplante, gestantes, portadores de neoplasias malignas, dependentes de álcool ou com fibrilação atrial.

Este trabalho foi aprovado pelo comitê de ética em pesquisa local (número 29999520.8.0000.5411), e o termo de consentimento livre e esclarecido foi dispensado.

As seguintes informações foram extraídas dos prontuários médicos dos pacientes: data da MAPA, idade, sexo, peso, altura, índice de massa corporal (IMC) e status tabágico.

As variáveis da MAPA registradas pelo dispositivo Spacelabs 90202 incluíram: PAS e PAD média nas 24 horas, durante o período de vigília e durante o sono. Com base nesses valores, foi calculada a queda noturna da PAS e diastólica. A queda noturna foi definida como uma redução da PA durante o sono entre 10% e 20% em relação aos níveis durante a vigília. A queda foi classificada como atenuada quando a redução variou entre 0% e 10%, e como ausente quando a PA noturna foi superior à diurna (ou seja, <0%).

### Análise estatística

As variáveis categóricas foram expressas em números absolutos e porcentagens. As variáveis contínuas foram submetidas a testes de normalidade e expressas como média ± desvio padrão, sendo utilizado o teste de Kolmogorov-Smirnov para avaliação da normalidade. As variáveis contínuas com distribuição normal foram comparadas por meio do teste t para amostras independentes. As variáveis com distribuição não normal foram comparadas utilizando o teste de Mann-Whitney. As variáveis categóricas foram comparadas pelo teste do qui-quadrado ou pelo teste exato de Fisher, quando apropriado. Foi realizada análise de regressão de Cox, considerando o óbito por todas as causas como desfecho primário e o óbito por causas cardiovasculares como desfecho secundário. A capacidade da queda noturna da PAS e PAD em prever os desfechos foi comparada. A análise de Cox foi ajustada para a PAS nas 24 horas. No modelo totalmente ajustado, foram incluídas as variáveis idade, diabetes, tabagismo, doença vascular aterosclerótica e doença renal crônica. Os resultados foram considerados significativos para p < 0,05. As análises estatísticas foram realizadas utilizando o software IBM SPSS Statistics versão 20.

## Resultados

Entre 27 de janeiro de 2004 e 16 de fevereiro de 2012, foram realizados um total de 1308 MAPAs. Desses, 52 foram considerados tecnicamente inadequados, 92 eram de pacientes com menos de 18 anos, 18 de receptores de transplante renal, 149 eram duplicatas, 129 apresentavam informações insuficientes e 112 eram de pacientes em diálise. Esses exames foram excluídos da análise. Assim, a coorte final foi composta por 756 pacientes submetidos ao MAPA de 24 horas. Dentre eles, 320 (42%) eram do sexo masculino, 710 (93,9%) eram caucasianos ou asiáticos, e 46 (6,1%) eram descendentes de africanos. A mediana de idade dos pacientes foi de 56 anos (intervalo interquartil: 42–68). Além disso, 217 pacientes (28,7%) eram diabéticos e 96 (13%) apresentavam doença aterosclerótica.

Dos 596 pacientes com dados disponíveis sobre tabagismo, 194 (25,6%) eram fumantes – 61 fumantes ativos e os demais ex-fumantes. A taxa de filtração glomerular mediana foi de 73 mL/min/1,73 m^2^ (intervalo interquartil: 48–95), e o IMC médio foi de 28 ± 5,9 Kg/m^2^.

Os resultados da MAPA mostraram que a queda da PAS durante o sono estava presente em 279 pacientes (36,9%), atenuada em 363 (48,0%) e ausente em 114 pacientes (15,1%). A queda da PAD estava presente em 453 pacientes (59,9%), atenuada em 227 (30,0%) e ausente em 76 pacientes (10,1%). A média da PAS em 24 horas foi de 130 ± 16,3 mmHg e a média da PAD foi de 78 ± 11,4 mmHg.

A duração média do acompanhamento foi de 106 meses, com mediana de 119 meses, variando de 1 a 206 meses. Durante esse período, ocorreram 95 eventos fatais, dos quais 27 foram atribuídos a causas cardiovasculares.

Os pacientes que apresentaram desfecho fatal (n=95) tiveram valores mais baixos de PAD nas 24 horas, PAD durante a vigília e queda da PAD. Não foram observadas diferenças estatisticamente significativas em relação à PAS nas 24 horas, PAS durante a vigília ou PAS durante o sono, embora valores numericamente mais elevados tenham sido registrados entre os pacientes que tiveram desfecho fatal. Também não houve diferença estatística na PAD durante o sono e na queda da PAS. Esses dados estão apresentados na Tabela 1.

**Tabela 1:** Dados da monitorização ambulatorial da pressão arterial estratificados pela ocorrência de eventos fatais por qualquer causa

Variável	Desfecho fatal		p
	Sim (95)	Não (661)	
PAS 24h (mmHg)	130±18,6	129±16,0	0,629
PAD 24h (mmHg)	74±11,8	78±11,3	<0,001
PAS durante a vigília (mmHg)	133±18,6	132±16,1	0,818
PAS durante o sono (mmHg)	126±19,9	123±18,4	0,135
PAD durante a vigília (mmHg)	76±11,9	81±11,8	<0,001
PAD durante o sono (mmHg)	69±12,5	71±12,1	0,134
Queda da PAS (%)*	6,4 (-8,9-21,7)	7,9 (3,2-11,1)	0,069
Queda da PAD (%)*	8,4 (2,5-10,9)	12,3 (6,5-18,1)	0,011

PAS: pressão arterial sistólica; PAD: pressão arterial diastólica;

* mediana (1ºquartil-3ºquartil).

Entre os 27 pacientes que apresentaram desfecho cardiovascular fatal, foram observadas diferenças estatísticas – maior PAS durante o sono, e reduções menores na queda da PAS e da PAD. Não foram encontradas diferenças estatisticamente significativas na PAS de 24 horas, PAD de 24 horas, PAS durante a vigília, PAD durante a vigília ou PAD durante o sono em comparação com os demais pacientes. Esses dados estão apresentados na Tabela 2.

**Tabela 2:** Dados da monitorização ambulatorial da pressão arterial estratificados pela ocorrência de eventos cardiovasculares

Variável	Desfecho cardiovascular fatal		p
	Sim (27)	Não (729)	
PAS 24h (mmHg)	134±17,9	129,5±16,27	0,142
PAD 24h (mmHg)	75±15,05	78±11,30	0,268
PAS durante a vigília (mmHg)	136±17,4	132±16,34	0,269
PAS durante o sono (mmHg)	130±20,5	123±18,4	0,036
PAD durante a vigília (mmHg)	77±15	81±12	0,083
PAD durante o sono (mmHg)	72±15,8	71±12	0,767
Queda da PAS (%)*	4,0 (-11,0-19,0)	7,7 (3,0-12,3)	0,028
Queda da PAD (%)*	6,8 (-0,4-14,0)	12,1 (6,3-17,9)	0,004

PAS: pressão arterial sistólica; PAD: pressão arterial diastólica;

* mediana (1ºquartil-3ºquartil).

Na análise de Cox univariada, a queda da PAS não esteve associada à mortalidade por todas as causas. No entanto, a ausência de queda da PAD esteve significativamente associada ao desfecho primário (HR: 2,08; IC95%: 1,18–3,69), em comparação com a presença de queda fisiológica da PAD. Na análise de regressão de Cox múltipla, ajustada para a PAS de 24 horas, apenas a ausência de queda da PAD manteve associação significativa com a mortalidade por todas as causas (HR: 2,05; IC95%: 1,15–3,67). No modelo totalmente ajustado – que incluiu idade, diabetes, tabagismo, doença vascular aterosclerótica e doença renal crônica – tanto a PAS quanto a PAD perderam a associação estatisticamente significativa. Esses dados estão apresentados na Tabela 3.

**Tabela 3:** Associação entre a queda noturna da pressão arterial sistólica (PAS) e da pressão arterial diastólica (PAD) e a mortalidade por todas as causas; análise de Cox univariada e ajustada para a PAS de 24 horas

		Univariada				Ajustada para PAS de 24 horas				Totalmente ajustada*			
		HR	IC 95%		p	HR	IC95%		p	HR	IC95%		p
			Inferior	Superior			Inferior	Superior			Inferior	Superior	
**Queda da PAS**													
	**Presente**	**Referência**				**Referência**				**Referência**			
	**Atenuada**	1,43	0,89	2,28	0,13	1,43	0,89	2,27	0,14	1,01	0,48	2,14	0,97
	**Ausente**	1,68	0,94	3,01	0,08	1,64	0,91	2,69	0,10	0,65	0,25	1,64	0,36
**Queda da PAD**													
	**Presente**	**Referência**				**Referência**				**Referência**			
	**Atenuada**	1,24	0,77	2,00	0,38	1,23	0,76	1,99	0,39	1,17	0,59	2,31	0,65
	**Ausente**	2,08	1,18	3,69	0,01	2,05	1,15	3,67	0,01	0,88	0,35	2,26	0,80

* Ajustado para idade, presença de diabetes mellitus, tabagismo, doença vascular aterosclerótica e doença renal crônica.

Na análise de Cox univariada para mortalidade cardiovascular, a ausência de queda da PAS esteve associada a esse desfecho (HR: 3,26; IC95%: 1,13–9,40). A ausência de queda da PAD também esteve associada ao desfecho secundário (HR: 3,80; IC95%: 1,53–9,44). Na análise de regressão de Cox múltipla, ajustada para a PAS de 24 horas, apenas a ausência de queda da PAD esteve associada ao aumento do risco cardiovascular (HR: 3,33; IC95%: 1,32–8,41), em comparação com a presença de queda fisiológica noturna. A ausência e a atenuação da queda da PAS não apresentaram associação estatisticamente significativa com a ocorrência dos desfechos quando ajustadas para a PAS de 24 horas.

No modelo totalmente ajustado, que incluiu idade, diabetes, tabagismo, doença vascular aterosclerótica e doença renal crônica, tanto a PAS quanto a PAD perderam a associação estatisticamente significativa. Esses dados estão apresentados na Tabela 4.

**Tabela 4:** Associação entre a queda noturna da pressão arterial sistólica (PAS) e da pressão arterial diastólica (PAD) e a mortalidade por doenças cardiovasculares; análise de Cox univariada e ajustada para a PAS de 24 horas

		Univariada				Ajustada para PAS de 24 horas				Totalmente ajustada*			
		HR	IC 95%		p	HR	IC95%		p	HR	IC95%		p
			Inferior	Superior			Inferior	Superior			Inferior	Superior	
**Queda da PAS**													
	**Presente**	**Referência**				**Referência**				**Referência**			
	**Atenuada**	1,7	0,67	4,67	0,25	1,75	0,66	4,61	0,26	1,01	0,48	2,14	0,97
	**Ausente**	3,2	1,13	9,40	0,03	2,8	0,98	8,40	0,0	0,65	0,25	1,64	0,36
**Queda da PAD**													
	**Presente**	**Referência**				**Referência**				**Referência**			
	**Atenuada**	1,22	0,47	3,17	0,68	1,15	0,44	3,00	0,78	2,00	0,41	9,68	0,39
	**Ausente**	3,80	1,53	9,44	<0,01	3,33	1,32	8,41	0,01	2,57	0,23	28,64	0,44

* Ajustado para idade, presença de diabetes mellitus, tabagismo, doença vascular aterosclerótica e doença renal crônica.

A Figura Central apresenta um resumo dos resultados.

## Discussão

Com o objetivo de comparar o desempenho da queda da PAS com a queda da PAD na predição de desfechos clínicos, foi analisada uma coorte de 756 pacientes submetidos à MAPA de 24 horas. A análise univariada revelou que a ausência de queda da PAD esteve associada à mortalidade por todas as causas e a desfechos cardiovasculares fatais, enquanto a ausência de queda da PAS esteve associada apenas aos desfechos cardiovasculares. Na análise múltipla, apenas a ausência de queda da PAD demonstrou poder preditivo para os desfechos primário e secundário, alcançando significância estatística mesmo após ajuste para a PAS de 24 horas. No modelo totalmente ajustado, nem a PAS nem a PAD apresentou associação estatisticamente significativa com qualquer um dos desfechos.

A MAPA não apenas fornece registros da PA ao longo de 24 horas, como também é o único método capaz de avaliar a PA durante o sono, permitindo a análise dos padrões de queda noturna. Grandes estudos de coorte^5-7^ e metanálises^8,9^ demonstraram que a presença de queda noturna – tanto da PAD quanto da PAS – está associada a um prognóstico mais favorável em comparação com sua ausência.

No entanto, em nosso conhecimento, poucos estudos avaliaram se o impacto da queda da PAS é maior do que o da PAD, ou vice-versa. Um estudo^9^ relatou que, em idosos, a queda da PAS prevaleceu em relação à queda da PAD, destacando a importância da queda noturna da pressão arterial. Nossos achados corroboram essa importância, uma vez que foi observado um maior risco de desfechos fatais na ausência da queda.

A explicação para esses resultados está nos mecanismos fisiológicos que ocorrem durante o sono, nos quais o sistema nervoso autônomo desempenha um papel central. O sono é composto por duas principais fases: REM (movimento rápido dos olhos) e não-REM (sem movimento rápido dos olhos). A fase não-REM é particularmente importante para a regulação da pressão arterial, pois é caracterizada pela predominância da atividade parassimpática. Isso ocorre devido ao aumento do tônus vagal, que reduz a frequência cardíaca e o débito cardíaco. Como resultado, a PA diminui naturalmente durante essa fase.^10^ Qualquer alteração nesse mecanismo pode comprometer o padrão normal de queda da pressão arterial, levando potencialmente ao aumento da pressão durante o sono. Essa alteração está associada a um maior risco de lesão em órgãos-alvo e doenças cardiovasculares, sendo comumente relacionada a condições como diabetes, hipertensão secundária, doenças arteriais de diversas etiologias, com destaque para a apneia obstrutiva do sono.^11^

As alterações na PAD são predominantemente influenciadas por modificações na resistência periférica, enquanto as alterações na PAS são principalmente decorrentes de variações no débito cardíaco.^12^ Esses mecanismos fisiológicos podem ajudar a explicar os resultados deste estudo, no qual a queda da PAD apresentou uma associação mais forte com os desfechos clínicos do que a queda da PAS.

Estudos anteriores^13-15^ associaram o aumento da PAD a um risco cardiovascular mais elevado em comparação ao aumento da PAS, especialmente em pacientes mais jovens.^13^ Isso ressalta a importância da PAD e de variáveis relacionadas, como a queda noturna, na predição da mortalidade. É importante destacar que nosso estudo não investigou a significância prognóstica dos valores médios de PAS ou PAD, mas sim a relevância prognóstica dos padrões de queda noturna dessas pressões. Outro estudo^16^ identificou que a ausência de queda, tanto da PAD quanto da PAS, esteve associada a desfechos fatais. Por outro lado, observou-se uma correlação mais forte entre eventos cardiovasculares e a queda da PAD, enquanto a ausência de queda da PAS esteve mais relacionada a eventos não cardiovasculares. Nosso estudo reforça a hipótese de que a ausência de queda da PAD está especificamente associada ao risco cardiovascular.

Por fim, neste estudo, foram controlados outros fatores de confusão além da pressão arterial, como idade, diabetes mellitus, tabagismo, doenças vasculares ateroscleróticas e doenças renais crônicas. Após o ajuste completo, a significância estatística das associações entre a queda da PAS e da PAD com os desfechos foi perdida. Esse achado sugere que a associação entre a queda da PA durante o sono e os desfechos pode ser mediada por esses fatores. No entanto, isso não diminui a importância de nossos achados, uma vez que nosso objetivo principal foi avaliar a significância prognóstica da ausência de queda, e não seu papel patogênico.

Este estudo observacional e retrospectivo apresenta algumas limitações. Trata-se de um estudo unicêntrico, o que limita a validade externa dos achados. Além disso, o número limitado de desfechos restringiu a possibilidade de realizar análises múltiplas com uma gama mais ampla de variáveis de confusão. A composição étnica da amostra também difere das distribuições populacionais global e brasileira.

No entanto, o tamanho da amostra foi adequado para atender aos objetivos propostos. Além disso, o fato de todos os pacientes terem sido acompanhados pela mesma equipe aumenta a reprodutibilidade dos resultados e fortalece a validade interna do estudo. Por fim, o foco exclusivo em eventos fatais ajuda a minimizar vieses subjetivos e evita a inclusão de pacientes com diagnósticos cardiovasculares imprecisos.

## Conclusão

A ausência de queda da PAD mostrou associação estatisticamente significativa com os desfechos clínicos, mesmo após o ajuste para a PAS média de 24 horas. Em contraste, a ausência de queda da PAS esteve significativamente associada apenas aos desfechos cardiovasculares, e somente quando não ajustada para a PAS média de 24 horas. Portanto, nossos achados indicam que a queda da PAD durante o sono possui maior poder preditivo para desfechos clínicos do que a queda da PAS, conforme ilustrado na Figura Central.

## Disponibilidade de Dados

Os conteúdos subjacentes ao texto da pesquisa estão contidos no manuscrito.
