# Supplementary material for: Queda da Pressão Arterial Diastólica Durante o Sono tem Maior Poder Preditivo Não Ajustado em Comparação à Queda Sistólica: Um Estudo de Coorte Retrospectivo
Source: Arq Bras Cardiol. 2025 Nov 18;122(10):e20250305. [Article in Portuguese] doi: 10.36660/abc.20250305 (PMC12671603; doi:10.36660/abc.20250305)
Supplement: Translation

# Translation: Diastolic Blood Pressure Dipping During Sleep Shows Superior Unadjusted Predictive Power Compared to Systolic Dipping: A Retrospective Cohort Study

**DOI:** 10.36660/abc.20250305i

**Authors:** Mateus de Carvalho Gonçalves, Daniely Santos da Silva, Vitória Nogueira Ribeiro, Vanessa Burgugi Banin, Vanessa dos Santos Silva, Rodrigo Bazan, Silméia Garcia Zanati Bazan, Pasqual Barretti, Luis Cuadrado Martin

**Affiliations:** 1 Botucatu SP Brasil Universidade Estadual Paulista Júlio Mesquita Filho - Faculdade de Medicina, Botucatu, SP – Brasil

**Keywords:** Hypertension, Ambulatory Blood Pressure Monitoring, Mortality

## Abstract

**Background:**

Sleep blood pressure (BP) fall is a prognostic marker. Few studies have evaluated whether systolic BP (SBP) or diastolic BP (DBP) dipping more accurately predicts clinical outcomes.

**Objective:**

To determine which type of BP dipping has greater predictive value for clinical outcomes.

**Methods:**

This retrospective cohort evaluated patients who underwent ambulatory blood pressure monitoring (ABPM) between January 27, 2004, and February 16,2012. Patients were followed until the occurrence of the primary outcome (death from any cause), or until the end of follow-up period (February 01, 2022). Cox survival curves were constructed to assess which dipping classification – SBP or DBP – better distinguished the occurrence of outcomes. Dipping was defined as a nocturnal BP reduction of 10-20%. Absent and attenuated dipping were defined as reductions of ≤ 0% and 0-10%, respectively. Statistical significance was set at p < 0.05.

**Results:**

A total of 756 patients were included, with a mean age of 54±16.4 years; 42% were male. In predicting the primary outcome (all-cause mortality), the absence of DBP dipping, adjusted for 24-hour SBP, was associated with a hazard-ratio (HR) of 2.051 (95% confidence interval [95%CI]: 1.147 - 3.670 (p=0.015). For the secondary outcome (cardiovascular mortality), the absence of DBP dipping, also adjusted for 24-hour SBP, showed a HR of 3.329 (95%CI: 1.317-8.412; p=0.011). In contrast, the absence of SBP dipping, when adjusted for 24-hour SBP, was not significantly associated with either outcome. In the fully adjusted model, which included age, diabetes, smoking, atherosclerotic vascular disease and chronic kidney disease, both SBP and DBP dipping lost statistically significant associations.

**Conclusion:**

DBP dipping demonstrates superior predictive power for outcomes compared to SBP dipping, and adds to the prognostic value of mean 24-hour SBP. (Central Illustration).

## Introduction

It is estimated that more than one billion people in the world have hypertension (1.39 billion in 2010).^1^ Among these individuals, a large proportion are unaware of their diagnosis and are not taking measures to control this chronic disease. This increases the chances of developing cardiovascular diseases,^2^ such as myocardial infarction, stroke, and chronic kidney disease, which collectively cause nearly 17.9 million deaths^3^ globally each year.

These data underscore the public health relevance of hypertension and the need for reliable methods of disease identification. Since blood pressure (BP) fluctuates with each heartbeat,^4^ diagnostic and therapeutic decisions cannot be made based on a single measurement. Access to daily average BP reading is essential, as some individuals may exhibit hypertensive levels during medical appointments, while having normal levels in their daily life (white coat hypertension). Conversely, others may show normal BP during appointments but experience elevated levels in daily life, referred to as masked hypertension. These discrepancies can only be accurately detected outside the clinical setting.

Out-of-clinic BP measurement methods include ambulatory blood pressure monitoring (ABPM),^4^ which is capable of measuring BP during both wakefulness and sleep. This test also indicates whether or not there is a significant physiological decrease (called dipping) in BP during sleep. The presence of nocturnal dipping is defined as a reduction in BP between 10% and 20% of daytime BP. This decrease is associated with better long-term outcomes regardless of the mean 24-hour BP.

When analyzing ABPM results, it is necessary to consider whether dipping is absent, attenuated, present, or accentuated (defined as less than 0%, less than 10%, between 10% and 20%, and greater than 20%, respectively). This classification may differ between systolic blood pressure (SBP) and diastolic blood pressure (DBP) and could show a physiological reduction in both DBP and SBP, a reduction only in DBP, only in SBP, or neither a reduction in DBP nor a reduction in SBP. Thus, the Brazilian ABPM guideline^4^ recommends that reductions in SBP and DBP be described separately in the examination report.

However, few studies have evaluated which type of dipping, SBP or DBP, more accurately predicts the future occurrence of clinical outcomes. The Brazilian guidelines^4^ even consider the level of evidence for this recommendation as "D", indicating it is based on expert opinion without conclusive evidence.

Therefore, this study aimed to determine whether - SBP dipping, DBP dipping, or both - has better predictive power for the occurrence of fatal events.

## Methods

A retrospective cohort study was conducted to determine whether the discriminatory power for predicting mortality in arterial hypertension differs between SBP dipping and DBP dipping during sleep. Hypertensive patients attending routine clinical follow-up, aged older than 18 years, and who underwent a single 24-hour ABPM between January 27, 2004, and February 16, 2012, were included.

ABPM was performed using appropriately sized cuffs for each patient and adhered to validation criteria: 16 daytime measurements and 8 nighttime measurements, taken at 15-minute intervals while awake and 30-minute intervals during sleep. Sleep and wakefulness periods were defined by the patients themselves, and monitoring was conducted on a typical day.

Follow-up began at the time of the first 24-hour ABPM recorded in the medical records. Patients were followed until the occurrence of the primary outcome (death from any cause, assessed through medical records or death certificates) or until the end of the follow-up period (02/01/2022). The secondary outcome was cardiovascular mortality (defined as death due to sudden cardiac, ischemic heart disease, heart failure, cerebrovascular disease, or aortic aneurysm). Patients with Alzheimer's or Parkinson's disease, those undergoing hemodialysis, transplant recipients, pregnant women, individuals with malignant neoplasms, alcohol dependance, or atrial fibrillation were excluded.

This work was approved by the local research ethics committee (number 29999520.8.0000.5411), and informed consent was waived.

The following information was extracted from patients’ medical records: date of ABPM date, age, sex, weight, height, body mass index (BMI), and smoking status.

ABPM variables recorded from the Spacelabs 90202 device included: mean SBP, DBP over 24 hours, during wakefulness, and during sleep. Based on these values, nocturnal dipping in systolic and DBP was calculated. Nocturnal dipping was defined as a reduction in BP during sleep of 10% to 20% compared to wakefulness levels. Nocturnal dipping was classified as attenuated when the reduction ranged from 0% to 10%, and absent when the nighttime BP exceeded daytime values (i.e., <0%).

### Statistical analysis

Categorical variables were expressed as absolute numbers and percentages. Continuous variables were subjected to normality tests and expressed as mean ± standard deviation, and the Kolmogorov-Smirnov test was used for the normality. Continuous variables with normal distribution were compared using the t-test for independent samples. Variables with non-normal distributions were compared using the Mann-Whitney test. Categorical variables were compared by chi-square or Fisher's exact test, when appropriate. Cox regression analysis was performed, considering death from all causes as the primary outcome and death from cardiovascular causes as the secondary outcome. The ability of nocturnal SBP and DBP dipping to predict the outcomes were compared. Cox analysis was adjusted for 24-hour SBP. In the fully adjusted model, age, diabetes, smoking, atherosclerotic vascular disease and chronic kidney disease were included. Results were considered significant for p < 0.05. The IBM SPSS Statistics 20 software was used for statistical analyses.

## Results

Between January 27, 2004, and February 16, 2012, a total of 1,308 ABPMs were performed. Of these, 52 were technically inadequate, 92 were from patients under 18 years of age, 18 were from kidney transplant recipients, 149 duplicates, 129 lacked sufficient patient information, and 112 were from patients undergoing dialysis. These were excluded from the analysis. As a result, the final cohort comprised 756 patients who underwent 24-h ABPM. Among them, 320 (42%) were male, 710 (93.9%) were Caucasian or Asian, and 46 (6.1%) were African descent. Patient median age was 56 (42-68) years. Additionally, 217 patients (28.7%) had diabetes, and 96 (13%) presented with atherosclerotic disease.

Of the 596 patients with available data on smoking, 194 (25.6%) were smokers (61 active smokers and the remainder were former smokers). The median glomerular filtration rate was 73 mL/min/1.73 m^2^ (interquartile range 48-95), and the mean BMI was 28 ± 5.9 Kg/m^2^.

ABPM results showed that SBP dipping was present in 279 patients (16.4%), attenuated in 363 (47.4%), and absent in 124 patients (16.2%). DBP dipping was present in 453 (59.1%), attenuated in 237 (30.9%), and absent in 76 patients (9.9%). The mean 24-h SBP was 130 ± 16.3 mmHg and the mean 24-h DBP was 78 ± 11.4 mmHg.

The mean follow-up duration was 106 months, with a median of 119 months, ranging from 1 to 206 months. During this period, 95 fatal events occurred, 27 of which were attributed to cardiovascular causes.

Patients who experienced a fatal outcome (n=95) had lower values for 24-h DBP, awake DBP, and DBP dipping. No statistically significant differences were observed in 24-h SBP, awake SBP, or sleep SBP, although numerically higher values were noted among patients who had a fatal outcome. There was also no statistical difference in sleep DBP and SBP dipping. These data are presented in Table 1.

**Table 1:** Ambulatory blood pressure monitoring data stratified by the occurrence of fatal events from any cause

Variable	Fatal outcome		p
	Yes (95)	No (661)	
24-hour SBP (mmHg)	130±18.6	129±16.0	0.629
24-hour DBP (mmHg)	74±11.8	78±11.3	<0.001
Awake SBP (mmHg)	133±18.6	132±16.1	0.818
Sleep SBP (mmHg)	126±19.9	123±18.4	0.135
Awake DBP (mmHg)	76±11.9	81±11.8	<0.001
Sleep DPB (mmHg)	69±12.5	71±12.1	0.134
SBP dipping (%)*	6.4(-8.9-21.7)	7.9(3.2-11.1)	0.069
DBP dipping (%)*	8.4(2.5-10.9)	12.3(6.5-18.1)	0.011

SBP: systolic blood pressure; DBP: diastolic blood pressure;

* median (1^st^ uartile-3^rd^ quartile).

Among the 27 patients who experienced a fatal cardiovascular outcome, statistical differences were observed: high sleep SBP, SBP dipping and DBP dipping was smaller. No statistically significant differences were found in 24-hour SBP, 24-hour DBP, awake SBP, awake DBP, or sleep DBP compared to the remaining patients. These data are presented in Table 2.

**Table 2:** Ambulatory blood pressure monitoring data stratified by the occurrence of cardiovascular events

Variable	Fatal cardiovascular outcome		p
	Yes (27)	No (729)	
24-hour SBP (mmHg)	134±17.9	129.5±16.27	0.142
24-hour DBP (mmHg)	75±15.05	78±11.30	0.268
Awake SBP (mmHg)	136±17.4	132±16.34	0.269
Sleep SBP (mmHg)	130±20.5	123±18.4	0.036
Awake DBP (mmHg)	77±15	81±12	0.083
Sleep DPB (mmHg)	72±15.8	71±12	0.767
SBP dipping (%)*	4.0(-11.0-19.0)	7.7(3.0-12.3)	0.028
DBP dipping (%)*	6.8(-0.4-14.0)	12.1(6.3-17.9)	0.004

SBP: systolic blood pressure; DBP: diastolic blood pressure;

* median (1^st^ quartile-3^rd^ quartile)

In the univariate Cox analysis, SBP dipping was not associated with all-cause mortality. However, the absence of DBP dipping was significantly associated with the primary outcome (HR: 2.08; 95%CI: 1.18-3.69) compared to the presence of physiological DBP dipping. In multiple Cox regression, adjusted for 24-h SBP, only the absence of DBP dipping was significantly associated with all-cause mortality (HR: 2.05; 95%CI: 1.15-3.67). In the fully adjusted model, which included age, diabetes, smoking status, atherosclerotic vascular disease and chronic kidney disease, both SBP and DBP lost their statistically significant association. These data are presented in Table 3.

**Table 3:** Association between nocturnal systolic blood pressure (SBP) and diastolic blood pressure (DBP) dips and all-cause mortality; univariate and adjusted Cox analysis for 24-hour SBP

		Univariate				Adjusted for 24h SBP				Fully Adjusted*			
		HR	95%CI		p	HR	95%CI		p	HR	95%CI		p
			Lower	Upper			Lower	Upper			Lower	Upper	
**SBP dip**													
	**Present**	**Reference**				**Reference**				**Reference**			
	**Attenuated**	1.43	0.89	2.28	0.13	1.43	0.89	2.27	0.14	1.01	0.48	2.14	0.97
	**Absent**	1.68	0.94	3.01	0.08	1.64	0.91	2.69	0.10	0.65	0.25	1.64	0.36
**DBP dip**													
	**Present**	**Reference**				**Reference**				**Reference**			
	**Attenuated**	1.24	0.77	2.00	0.38	1.23	0.76	1.99	0.39	1.17	0.59	2.31	0.65
	**Absent**	2.08	1.18	3.69	0.01	2.05	1.15	3.67	0.01	0.88	0.35	2.26	0.80

* Adjusted for age, presence of diabetes mellitus, smoking, atherosclerotic vascular disease and chronic kidney disease.

In univariate Cox analysis for cardiovascular mortality, the absence of SBP dipping was associated with this outcome (HR: 3.26 95%CI: 1.13-9.40). The absence of DBP dipping was also associated with the secondary outcome (HR: 3.80; 95%CI: 1.53-9.44). In the multiple Cox regression, adjusted for 24-hour SBP, only the absence of DBP dipping was associated with increased cardiovascular risk (HR: 3.33; 95%CI: 1.32-8.41) compared to the presence of physiological nocturnal dipping. The absence and attenuation of SBP dipping showed no statistically significant association with the occurrence of outcomes when adjusted for 24-hour SBP.

In the fully adjusted model, which included age, diabetes, smoking status, atherosclerotic vascular disease and chronic kidney disease), both SBP and DBP lost their statistically significant associations. These data are presented in Table 4.

**Table 4:** Association between nocturnal SBP and DBP dips and cardiovascular mortality. Univariate and adjusted Cox analysis for 24h SBP

		Univariate				Adjusted for 24h SBP				Fully Adjusted*			
		HR	95%CI		p	HR	95%CI		p	HR	95%CI		p
			Lower	Upper			Lower	Upper			Lower	Upper	
**SBP dip**													
	**Present**	**Reference**				**Reference**				**Reference**			
	**Attenuated**	1.7	0.67	4.67	0.25	1.75	0.66	4.61	0.26	1.01	0.48	2.14	0.97
	**Absent**	3.2	1.13	9.40	0.03	2.8	0.98	8.40	0.0	0.65	0.25	1.64	0.36
**DBP dip**													
	**Present**	**Reference**				**Reference**				**Reference**			
	**Attenuated**	1.22	0.47	3.17	0.68	1.15	0.44	3.00	0.78	2.00	0.41	9.68	0.39
	**Absent**	3.80	1.53	9.44	<0.01	3.33	1.32	8.41	0.01	2.57	0.23	28.64	0.44

* Adjusted for age, presence of diabetes mellitus, smoking, atherosclerotic vascular disease and chronic kidney disease.

Central Illustration presents a summary of the results on the cover.

## Discussion

In order to compare the performance of SBP dipping with the DBP dipping in predicting clinical outcomes, a cohort of 756 patients who underwent 24-hour ABPM was analyzed. The univariate analysis revealed that the absence of the DBP dipping was associated with all-cause mortality and fatal cardiovascular outcomes, whereas the absence of the SBP dipping was associated only with cardiovascular outcomes. Upon multiple analysis, only the absence of DBP dipping showed predictive power for both primary and secondary outcomes, achieving statistical significance even when adjusted for 24-hour SBP. In the fully adjusted model, neither SBP, nor DBP showed a statistically significant association with any outcome.

ABPM not only provides BP readings over a 24-hour period, but it is also the only method capable of assessing BP during sleep, thereby offering insights into nocturnal dipping patterns. Large cohort studies^5-7^ and meta-analyses^8,9^ have demonstrated that the presence of nocturnal dipping – both in DBP and SBP – is associated with a more favorable prognosis compared to its absence.

However, to our knowledge, few studies have assessed whether the impact of SBP dipping is greater than that of DBP dipping or vice versa. One study^9^ reported that, in the elderly, SBP dipping prevailed compared to DBP dipping, and highlighted the importance of nocturnal BP dipping. Our findings corroborate this importance as a greater risk of fatal outcomes was observed when the dip was absent.

The explanation for these results lies in the physiological mechanisms that occur during sleep, where the autonomic nervous system plays a central role. Sleep consists of two main stages – REM (rapid eye movement) and non-REM (non-rapid eye movement) sleep. The non-REM stage is particularly important for BP regulation, as it is characterized by a predominance of parasympathetic activity. This is due to an increase in vagal tone, which slows the heart rate and reduces cardiac output. As a result, BP naturally decreases during this stage.^10^ Any disruption in this mechanism can impair the normal dipping pattern, potentially leading to elevated BP during sleep. This is associated with a higher risk of target organ damage and cardiovascular disease and is commonly linked to conditions like diabetes, secondary hypertension, arterial diseases of various etiologies, notably obstructive sleep apnea.^11^

Changes in DBP are predominantly influenced by modifications in peripheral resistance, whereas changes in SBP are primarily driven by alterations in cardiac output.^12^ These physiological mechanisms may help explain the results of this study, in which DBP dipping showed a stronger association with clinical outcomes than SBP dipping.

Previous studies^13-15^ have linked elevated DBP to a higher risk of cardiovascular events compared to elevated SBP, particularly in younger patients.^13^ This highlights the importance of DBP and related variables, including nocturnal dipping, in predicting mortality. It is important to note that our study did not investigate the prognostic significance of mean of SBP or DBP values, but rather focused on the prognostic relevance of their nocturnal dipping patterns. Another study^16^ found that the absence of dipping, in both DBP and SBP, was associated with fatal outcomes. Interestingly, a stronger correlation was observed between cardiovascular events and DBP dipping, while the absence of SBP dipping was more closely correlated with non-cardiovascular events. Our study supports the hypothesis that the absence of DBP dipping is specifically associated with cardiovascular risk.

Finally, in this study, additional confounding factors beyond BP were controlled for, namely age, diabetes mellitus, smoking, atherosclerotic vascular diseases and chronic kidney diseases. After full adjustment, the statistical significance of the associations between both SBP and DBP dipping and outcomes was lost. This finding suggests that the association between sleep dipping and outcomes may be mediated by these factors. However, this does not diminish the importance of our findings, as our primary goal was to assess the prognostic significance of the absence of dipping, rather than its pathogenic role.

There are some limitations to this observational and retrospective study. This is a single-center study, which limits the external validity of the findings. In addition, the limited number of outcomes restricted the ability to perform multiple analyses with a broader range of confounding variables. The ethnic composition of the sample differs from both global and Brazilian population distributions.

However, the sample size was adequate to address the proposed objectives. Additionally, the fact that all patients were followed by the same team enhances the reproducibility of the results and strengthens the internal validity of the work. Finally, focusing exclusively on fatal events helps minimize subjective bias and avoids the inclusion of patients with potentially inaccurate cardiovascular diagnoses.

## Conclusion

The absence of DBP dipping showed a statistically significant association with clinical outcomes, even after adjustment for mean 24-hour SBP. In contrast, the absence of SBP dipping was significantly associated only with cardiovascular outcomes, and only when not adjusted for mean 24-hour SBP. Therefore, our findings indicate that DBP dipping during sleep has greater predictive power for clinical outcomes than SBP dipping, as illustrated in the central figure on the cover.

## Data Availability

The underlying content of the research text is contained within the manuscript.
